# Editorial: Ocular complications associated with diabetes mellitus

**DOI:** 10.3389/fendo.2023.1193522

**Published:** 2023-04-03

**Authors:** Xinyuan Zhang, Sobha Sivaprasad, Daniel Shu Wei Ting

**Affiliations:** ^1^ Beijing Tongren Eye Center, Beijing Tongren Hospital, Capital Medical University, Beijing, China; ^2^ Beijing Retinal and Choroidal Vascular Disorders Study Group, Beijing, China; ^3^ NIHR Moorfields Biomedical Research Centre, Moorfields Eye Hospital, London, United Kingdom; ^4^ Singapore Eye Research Institute, Singapore National Eye Center, Duke-NUS Medical School, National University of Singapore, Singapore, Singapore

**Keywords:** diabetic retinopathy, micro-vasculopathy, diabetes mellitus, ocular surface, prevention and intervention, artificial intelligence

Diabetes mellitus (DM) is a major expanding health problem with the fastest growing health challenges in the 21^st^ century. DM is strongly associated with both microvascular and macrovascular complications. The eye is a window to several systemic and neuro-ophthalmic complications and associations of diabetes (1). The retinal vascular fractals reflect long-term microvasculopathy and the pathological and morphological changes of corneal nerve reflect severity of diabetic neuropathy. Preventive measures and early intervention can significantly reduce the morbidity caused by ocular complications associated with diabetes.

This Research Topic highlight the wide spectrum of latest advancements in basic and clinical concepts in the diagnosis, progression, treatment outcomes, and the application of AI techniques in clinical research for diabetes related complications.

## Diabetic retinopathy

Diabetic retinopathy (DR) is the leading cause of blindness in the working age population in both developed and developing countries. DR and diabetic nephropathy (DKD) are the commonest microvascular complications of DM. Early diagnosis and prompt intervention of sight threatening DR are critical in patients with DM to achieve a good visual outcome. Molecular and imaging biomarkers and artificial intelligence (AI), as well as gene and stem cell research are gathering pace in both early prevention and management of DR. In this Research Topic, Tan and Wong discusses the major trends in DR in 2023 that includes epidemiology especially the global burden of DR, the pathophysiological understanding of DR especially retinal neural dysfunction and, the application of new imaging modalities and AI in DR. Xie and Xiao provide insight into the recent advancement and progress made on inflammatory and arteriosclerosis-associated biomarkers; novel therapeutic strategies including anti-VEGF therapy, renin-angiotensin-aldosterone system therapy, nanotechnology on DR and diabetic nephropathy (DKD). Zhang et al. investigate the mechanisms underlying the correlations between DR and DKD in patients with T2DM. The findings highlight the predictive role of Albumin-to-creatinine ratio on DR severity and progression, indicating the link between DR and DKD and the association with dyslipidemia and upregulated circulating level of angiogenic cytokines. Furthermore, Chen et al., Liu et al., Huang et al. describe potential circulating molecular biomarkers and targets for DR including Glucagon-like peptide-1 receptor (GLP-1R), sodium-glucose co-transporter (SGLT) 1, SGLT2, Glucose transporter type 1 (GLUT1) and GLUT2 ferroptosis-related, L-Citrulline, hexanoylcarnitine, chenodeoxycholic acid and eicosapentaenoic acid. By using a non-parametric technique, Wang et al. identified eight predictive risk factors for DR including disease duration, body mass index, fasting blood glucose, glycated hemoglobin homeostatic model assessment-insulin resistance, triglyceride, total cholesterol and vitamin D-T3. These interesting discoveries from bench or bedside will likely become important components of translational research.

Several AI techniques, such as machine learning and deep learning, have been applied in automated screening, diagnosis and prognosis prediction of DR and diabetic macular edema (DME). The integration of AI with imaging technologies such as digital fundus photography and optical coherent tomography will continue to be an important area of DR research, with the potential to further enhance our clinical practice. In this Research Topic,Sheng et al. highlighted the fundamental concepts of AI and its application in DR and further discuss the current challenges and prospects of AI in ophthalmology. A machine learning based and molecular docking methods were also applied to identify the potential ferroptosis-related biomarkers and pharmacological compound in DR by Liu et al. An overview of global publications on machine learning in DR from 2011 to 2021 is also presented by Shao et al. They conclude that diverse and multiple modalities of medical data, new ML techniques and constantly optimized algorithms are the future research areas in DR.

## Diabetic ocular surface diseases

While DR is the most well-known complication of DM, ocular surface diseases, including dry eye disease (DED) and diabetic keratopathy are also common in the diabetic population. These diabetic ocular surface diseases may seriously affect the quality of life. Accumulation of advanced glycation end-products impaired neurotrophic innervation and limbal stem cell function, dysregulated growth factor signaling, and inflammation contribute to the pathogenesis of diabetic keratopathy. Lacrimal Functional Unit dysfunction, abnormal tear dynamics, and film dysfunction have been implicated in the pathogenesis of DED. In this Research Topic, Zhou et al. highlight the important roles of the dense innervations in the homeostatic maintenance of cornea and the lacrimal gland. The clinical manifestation, potential treatment options and underlying pathological mechanisms of diabetic keratopathy (diabetic corneal epitheliopathy and corneal neuropathy), diabetic corneal endotheliopathy, diabetic dry eye, diabetic meibomian gland dysfunction have been illustrated in detail. They further emphasize that studies on the neuroepithelial and neuroimmune interactions will likely reveal predominant pathogenic mechanisms and contribute to the development of intervention strategies of diabetic ocular surface complications. Liu et al. further provide an overview of the morphological changes of diabetic corneal neuropathy using *in-vivo* confocal microscopy in both animal and clinical studies. They introduce the pathological changes in maturation stages of corneal dendritic cells (DCs) in DM, emphasizing the relationship between corneal DCs and clinical parameters including age, corneal nerve status and metabolism parameters., The two comprehensive reviews provide valuable insight into the development of diagnostic, preventive, and therapeutic strategies for DM-associated ocular surface complications.

## Diabetes associated glaucoma

Glaucoma is a significant cause of blindness worldwide. Primary open angle glaucoma (POAG) is the most common type of glaucoma in patients with DM (2). The commonest type of secondary glaucoma in patients with DM in clinical practice is neovascular glaucoma (NVG), which is characterized by the appearance of neovascular over the iris and the proliferation of fibrovascular tissue in the anterior chamber angle mainly due to DR. In this Research Topic, Tang et al. outline the underlying mechanisms management strategies of NVG in patients with DM in eyes with proliferative DR (PDR). In a mini review article, Cheng et al. describe the correlations between biomechanical dyshomeostasis and glaucoma and other ocular diseases, providing novel diagnostic and treatment strategies targeting mechanobiology of these disorders.

## Summary

In conclusion, DM associated ocular complications has progressively and rapidly becoming the most significant cause of morbidity, which are preventable with early detection and timely management. Besides DR, diabetic ocular surface disorder and glaucoma, other DM associated ocular complications including cataract, DM related refractive changes, eye infection, optic neuropathies (diabetic papillopathy, non-arteritic anterior ischemic optic neuropathy) etc. can also be caused by chronic hyperglycemia. Routine eye examinations and intervention at the right point as well as systemic interventions including blood glucose, hypertension and hyperlipidemia control are essential for the reduction of DM related vision loss. The convergence of technologies and the proliferation of biologics as therapeutics promise to provide more novel and effective treatment options as augmentations or through other delivery methods. This Research Topic provided new insight into the mechanisms, molecular biomarkers, AI, and intervention strategies for DM associated ocular complications.

## Author contributions

XZ drafted and revised the manuscript, SS and DT provided comments and made revisions. All authors contributed to the article and approved the submitted version.
